# Supplementary material for: Revealing Genetic Differences in Fiber Elongation between the Offspring of Sea Island Cotton and Upland Cotton Backcross Populations Based on Transcriptome and Weighted Gene Coexpression Networks
Source: Genes (Basel). 2022 May 26;13(6):954. doi: 10.3390/genes13060954 (PMC9222338; doi:10.3390/genes13060954)
Supplement: Supplementary file 1 [file genes-13-00954-s001.zip › Supplementary material/Figure S1.Amino acid sequence alignment of 21 hub-genes in Upland cotton and Sea island cotton.pdf]

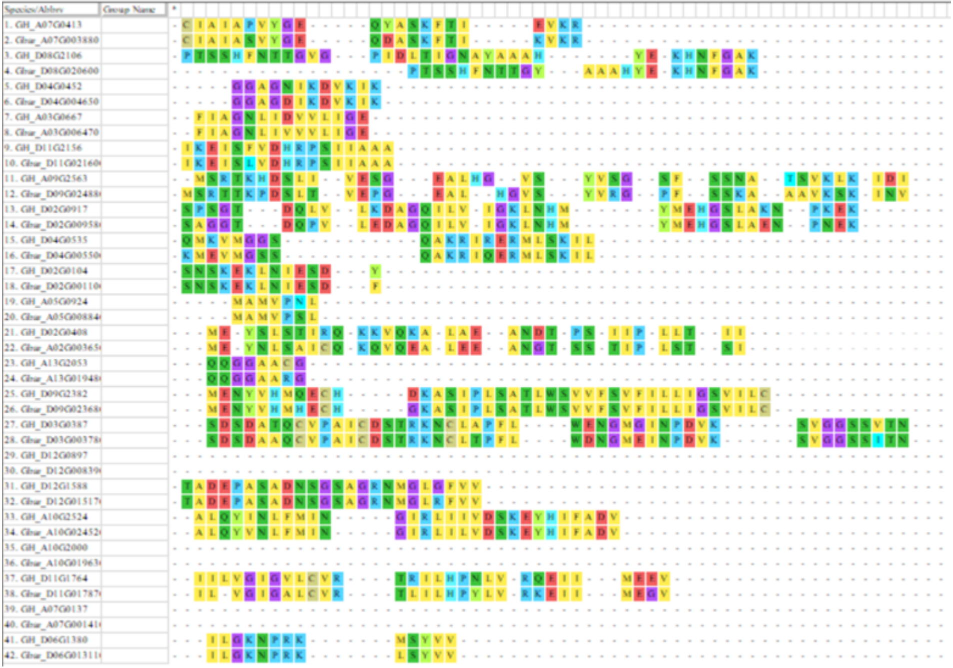
Species/Abbrv
Group Name
1. GH_A07G0413
2. Gbar_A07G003880
3. GH_D08G2106
4. Gbar_D08G020600
5. GH_D04G0452
6. Gbar_D04G004650
7. GH_A03G0667
8. Gbar_A03G006470
9. GH_D11G2156
10. Gbar_D11G02160
11. GH_A09G2563
12. Gbar_D09G02488
13. GH_D02G0917
14. Gbar_D02G00958
15. GH_D04G0535
16. Gbar_D04G00550
17. GH_D02G0104
18. Gbar_D02G00110
19. GH_A05G0924
20. Gbar_A05G00884
21. GH_D02G0408
22. Gbar_A02G00365
23. GH_A13G2053
24. Gbar_A13G01948
25. GH_D09G2382
26. Gbar_D09G02368
27. GH_D03G0387
28. Gbar_D03G00378
29. GH_D12G0897
30. Gbar_D12G00839
31. GH_D12G1588
32. Gbar_D12G01517
33. GH_A10G2524
34. Gbar_A10G02452
35. GH_A10G2000
36. Gbar_A10G01963
37. GH_D11G1764
38. Gbar_D11G01787
39. GH_A07G0137
40. Gbar_A07G00141
41. GH_D06G1380
42. Gbar_D06G01311
